# Facilitative Effects of Embodied English Instruction in Chinese Children

**DOI:** 10.3389/fpsyg.2022.915952

**Published:** 2022-07-14

**Authors:** Connie Qun Guan, Wanjin Meng

**Affiliations:** ^1^School of Foreign Studies, Beijing Language and Culture University, Beijing, China; ^2^Department of Psychology, Carnegie Mellon University, Pittsburgh, PA, United States; ^3^Department of Moral, Psychological and Special Education, China National Institute of Education Sciences, Beijing, China

**Keywords:** embodied cognition, language instruction, explicit morphological training, English as a second language, handwriting, gesture

## Abstract

Research into the lexical quality of word representations suggests that building a strong sound, form, and meaning association is a crucial first step for vocabulary learning. For children who are learning a second language (L2), explicit instruction on word morphology is generally more focused on whole word, rather than sub-lexical, meaning. Though morphological training is emphasized in first language (L1) vocabulary instruction, it is unknown whether this training facilitates L2 word learning through sub-lexical support. To test this, we designed three experimental learning conditions investigating embodied morphological instruction [i.e., hand writing roots (HR), dragging roots (DR), gesturing roots (GR)] to compare against a control condition. One hundred students were randomly assigned to the four experimental groups. Pre- and post-tests examining knowledge of word meanings, forms, and sounds were administered. Results of mixed linear modeling revealed that three embodied morphological instruction on roots enhanced L2 vocabulary learning. Hand writing roots facilitated sound-meaning integration in all category-tasks for accessibility to word form and one task for word sound-form association. By contrast, GR facilitated meaning-based learning integration in two out of three category tasks for word form-meaning association. Chunking and DR facilitated meaning-based integration in one out of three category tasks for word form-meaning association. These results provide evidence that the underlying embodied morphological training mechanism contributes to L2 vocabulary learning during direct instruction. Future directions and implications are discussed.

## Introduction

“Learning by doing” has long been recognized as an effective learning mechanism since the early 19^th^ century (Dewey and Authentic, 1938). It refers to learning from experiences resulting directly from one’s own actions (Reese, 2011). Learning to read through bodily engagement with the written form of language could be facilitatory for vocabulary acquisition for children (James, 2010; Kontra et al., 2012; Guan et al., 2021). Embodied learning is defined as the connection between bodily movements and cognitive abilities. Embodied learning shows the relation among movements and academic achievement; in other words, it is the relation of mind and body (Foglia and Wilson, 2013). Embodied language learning emphasizes the usage of motor or sensory movements to process and understand language material, verbs, nouns, or sentences (Buccino and Mezzadri, 2015). Word learning, especially action verbs, often co-occur with bodily movements or visual sensations, contributing to strengthening the link between sensorimotor programs and linguistic concepts (Vukovic and Shtyrov, 2014). The embodied experience might play a significant causal role in second language processing as well, in which a strong bond between context, sensory-motor experience and language was established (Pulvermüller and Fadiga, 2010).

Meaning-based instruction emphasizes the association among lexical constituents centered on the semantic representation of a word. However, contemporary teaching paradigms tend to focus more on phonological approaches such as repetition rather than paying explicit attention to lexical forms (De Jong et al., 2015). High-quality lexical representation requires efficient word recognition and strong association among lexical constituents such as form, sound, and meaning (Ehri, 2014; Stafura and Perfetti, 2014). In the native language acquisition process, mapping high-quality lexical and sub-lexical representations lays the foundation for subsequent acquisition of new words (Perfetti and Harris, 2013). Strong associations among these lexical constituents contribute to literacy in the first language (L1; Perfetti and Harris) and in a second language (L2; Gunderson and D’Silva, 2016). A critical, unresolved question is whether and to what extent explicit embodied instruction could lead to the sound-form-meaning association of high-quality lexical representations involving hand/body-related movements such as hand writing, chunking and dragging, and gesture. In the current study, we examine the question of how children learning English as a Second Language (ESL) can best use bodily engagement to enrich mental representations of the forms, meanings, and sounds of new vocabulary.

The goal of the current study is to provide empirical evidence for explicit embodied instruction in word learning in an L2 context. Presently, there is a wide variety of ways to transfer bodily engagement into learning (for a related discussion, see Skulmowski and Rey, 2018). A large part of embodied learning research is concerned with instructional techniques involving learners’ entire bodies (e.g., Johnson-Glenberg et al., 2014; Lindgren et al., 2016). However, other studies have focused on the potential uses of alternative embodied phenomena in educational contexts, including (1) gesturing (Goldin-Meadow, 2011; Pouw et al., 2014), (2) hand writing (De Koning and Tabbers, 2013; Wakefield and James, 2015), and (3) physically manipulating target objects during reading (Glenberg et al., 2013).

### Facilitating Letter and Word Recognition *via* Hand Writing

Hand writing facilitates visual word recognition and influences symbol learning by creating a network that includes both sensory and motor brain systems (Guan et al., 2011, 2015). Compared to non-motor practice, handwriting training produces faster learning and greater generalization to untrained tasks than previously reported. Handwriting practice leads to learning of both motor and symbolic letters (Wiley and Rapp, 2021). For example, hand writing Chinese characters improves Chinese second language learners’ ability to write Chinese characters and understand their spatial structure (Hsiao et al., 2015). Hand writing facilitates understanding of symbols by virtue of its environmental output, supporting the notion of developmental change through brain-body-environment interactions (Li and James, 2016). Neuroscience research has shown that brain mechanisms supporting visual letter categorization respond more strongly to letters after hand writing of those letters (Kersey and James, 2013). Handwritten letters can facilitate early letter comprehension due to the ability of handwriting to improve visual-motor coordination (Zemlock et al., 2018). Taken together, these results suggest that handwriting practice plays a key role in the brain networks underlying letter perception and lexical learning. However, previous studies have mostly explored the effects of hand writing in comparison with typing, viewing, etc. (Li and James, 2016; Zemlock et al., 2018), with few studies investigating whether hand writing associated with morphological structures at the sub-lexical level facilitate whole word learning.

### Facilitating Word Recognition *via* Chunking and Dragging

A *chunk* is defined as a sequence with the property that elements within the sequence, but not elements outside the sequence, predict each other (Cohen and Adams, 2001). Chunking refers to the organization and presentation of information into easily identifiable and manageable groups or units so that people can efficiently and effectively understand and process a message (Lorenz and Tizón-Couto, 2019). In cognitive psychology, chunking is a process by which individual pieces of a set of information are broken down and then combined into a meaningful whole. Information is grouped in large chunks to increase the short-term retention of material, thereby bypassing the limited capacity of working memory (Thalmann et al., 2019). Chunking, as a memory strategy, facilitates retention, word segmentation, reading, information processing, language acquisition, and comprehension (Gobet et al., 2001; Lorenz and Tizón-Couto, 2019). For example, reading skills depend in part on the development of higher-order perceptual units, sometimes referred to as chunking. This concept was proposed to help explain the so-called word comprehension effect, or the idea that more letters can be understood at once if they form a word than if they are unrelated (Stennett et al., 1973). Studies of Chinese character chunking have shown that while certain cortical areas of the dorsal and ventral pathways are activated during chunking, activation of early and higher visual cortical areas is inconsistent (Luo et al., 2006). Using fMRI to investigate the relationship between different frequencies and the chunk status of derived words (e.g., government, worthless), it was found that relative frequency affects the early stages of processing, thus supporting the notion of chunking based on the frequency of use elicited (Blumenthal-Dramé et al., 2017).

Several studies also support the idea that performing tracing activities with fingers and other simple hand movements can aid learning processes and language development (Freeman et al., 2011; Brooks and Goldin-Meadow, 2016). Researchers have measured hand movements on a screen to understand the dynamics of a broad range of psychological processes. Hand-tracking can provide unusually high-fidelity, real-time motor traces of the mind (Freeman et al., 2011). Spivey et al. (2005) asked participants to move the computer mouse from the bottom-center of the screen to the top-left or top-right corners, and hand movements showed a continuous attraction toward the distractor before settling into the correct alternative. Dragging as a kind of hand movement can facilitate word learning by dragging the individual chunks of letters or parts into words. From the perspective of motor skill development, one study tested participants on their use of click-drag-click or drag-and-drop motions and results showed that drag-and-drop task was faster and more accurate for children (Donker and Reitsma, 2007). Another study investigated whether point-and-click versus drag-and-drop interactions during game play had an effect on achievement and motivation (Inkpen, 2001) and results suggested that point-and-click was quicker, more accurate, and generally easier for children, and had a positive impact on motivation and success. Previous motor experience has been shown to affect how language is understood and processed—playing hockey can enhance one’s ability to understand language about hockey, apparently because brain areas normally used to perform an act become highly involved in understanding language about that act (Pulvermüller, 2005; Beilock et al., 2008). This study used physical manipulation (dragging) to help better understand the morphological structure and facilitate word recognition process. Gestalt psychology postulates that the perception of an entire string does not involve strong activation of its components, but that isolated components strongly evoke the whole.

Most studies on chunking and dragging have focused on L1 speakers (Perruchet et al., 2014); studies of chunking in L2 learners are relatively rare, tending to focus on the chunking effects of L1 and L2 segmentation processes (Mauranen, 2009; Franco and Destrebecqz, 2012). To date, very few studies investigate whether chunking and dragging of morphological structures at the sub-lexical level facilitates whole word learning.

### Facilitating L1 and L2 Learning *via* Gestures

Gestures are a form of communication in which body movements convey information supplementing information conveyed *via* language. Research on the role of gestures in the early stages of language learning has shown that pointing can be used to facilitate the expression of new ideas and words (Goldin-Meadow and Alibali, 2013). Moreover, gestures facilitate semantic processing of complex narratives in children as well as adults (Austin and Sweller, 2014; Dargue and Sweller, 2018). Gestures are closely associated with concomitant speech processing (Kelly et al., 2015) and are useful in disambiguation (Holle and Gunter, 2007) and communication (Kelly et al., 2015), with gestures conveying phonological information also playing a facilitating role (Holler and Wilkin, 2011). Research has shown that gestures can affect language comprehension in native speakers (Hostetter, 2011). With respect to second language learning, gestures can enhance L2 vocabulary acquisition and retention (Allen, 1995; Tellier, 2008; Kelly et al., 2009; Morett, 2014, 2018) and improve acquisition of novel L2 speech sounds (Morett and Chang, 2014; Zhen et al., 2019; Hoetjes and van Maastricht, 2020; Li et al., 2021). Gestures have been found to influence the three interrelated cognitive processes of communication, encoding, and recall for L2 vocabulary learning (Macedonia, 2014; Morett, 2014). Gestures accompanying L2 speech facilitate its acquisition by contributing to embodied representations, suggesting that gestures should be incorporated into L2 instruction as a learning tool (Macedonia, 2014). Because the majority of previous studies have focused on the phonological and communicative aspects of L2 learning in which gestures play a facilitatory role, little published research to date has investigated whether gestures associated with morphological structures at the sub-lexical level facilitate whole-word learning.

### Taxonomy

A large part of embodied learning research is concerned with instructional techniques involving learners’ entire bodies (Johnson-Glenberg et al., 2014; Lindgren et al., 2016). However, other studies have focused on the potential uses of alternative embodied phenomena in educational contexts, including (1) gesturing (Goldin-Meadow, 2011; Pouw et al., 2014), (2) hand writing (De Koning and Tabbers, 2013; Wakefield and James, 2015), and (3) physically manipulating target objects during reading (Glenberg et al., 2013).

Skulmowski and Rey (2018) have proposed a more general model based on the two dimensions of integration between bodily engagement and task performance. A 2 (levels of bodily engagement low vs. high) × 2 (levels of meaning association: low vs. high) grid was proposed according to the model/taxonomy to assess corresponding learning outcomes. Specifically, these are classified as follows: (1) hand writing (decoding + form + meaning mapping, and binding); (2) dragging (cue-based manipulation + form embedding); and (3) action (semantic + bodily engagement), as well as other embodied actions ranging from subtle finger typing to a global concept of learning by using the whole body. Their model posits that bodily activities could be integrated into learning tasks and that bodily action should be associated with enriched semantic representations of to-be-learned materials.

According to this taxonomy, the degree of the facilitative effect of instruction increases incrementally as the degree of bodily engagement increases. The current study implemented three types of experimental conditions, namely hand movement, and physical manipulation (i.e., chunking and dragging), and action. These conditions can be categorized as high integrated bodily engagement, low integrated bodily engagement, and low incidental bodily engagement, respectively. These three conditions allow us to compare embodied learning activities ranging from limited to full-body movement systematically and informatively. The activation of motor systems (body engagement or hand movement) facilitates semantic processing. Therefore, this experiment tested whether handwriting, dragging, and acting as embodied manipulative strategies in Latin root morphology training could improve child ESL learners’ vocabulary learning.

### Theories and Practices of Morphological Training

#### Current Model of Morphological Processing and Instruction

Morphosyntax is the system of rules that govern how morphemes are combined to form words that express different meanings. Morphemes are the smallest meaningful units in a given language, and include three morphemic patterns: inflectional affixes, derivational affixes, and roots. Morphological awareness is the metalinguistic awareness that words are made up of meaningful units. It is the foundation of generative word processing. Morphological awareness enables the development of effective and intrinsic morphological processing and problem-solving strategies. There is extensive evidence that morphological awareness is of great importance for word learning for both L1 and L2 English learners, as it is associated with literacy outcomes such as decoding (e.g., Singson et al., 2000), word identification (e.g., McCutchen et al., 2009), spelling ability (e.g., McCutchen and Stull, 2015), and reading comprehension (Crosson and McKeown, 2016).

The present study is based on Schreuder and Baayen’s (1995) theoretical model of morphological processing, which indicates how morphemes are established as memory representations. According to this theoretical model, form-meaning associations between orthographic strings and their corresponding meanings begin to be established *via* frequent encounters. Once learners discover the redundant form-meaning link, the memory representation (or concept node) is established. When the orthographic pattern is encountered again and again, the strength of the memory representation becomes stronger and stronger. In addition, when an unfamiliar morphologically complex word is encountered, representations of morphological structure can be activated in one’s memory. In this sense, this theoretical model demonstrates how morphemes can be used in morphological analysis to infer the meanings of unfamiliar words.

This theoretical model does not adequately account for the use of Latin roots in word learning. In the view of Schreuder and Baayen (1995), frequency and transparency are essential for activating and strengthening memory representations, but Latin roots are not always redundant and transparent enough to activate these representations. For example, some Latin roots are not transparent in semantics, which may affect their accessibility [e.g., The latin root “cal” refers to “to call,” but the word “calvary” (ordeal) has nothing to do with the meaning of “to call.” So sometimes the latin roots can be non-transparent]. Other Latin roots may have different phonological and orthographic forms, which may affect the accessibility of the morphological constituent. Therefore, the current framework merits further elaboration and extension to establish memory representations of Latin roots. Using morphological information about Latin roots to establish form-meaning associations and infer meanings of unfamiliar words has been overlooked in previous morphology research.

#### Latin Roots Training

Latin roots hold great value in facilitating L2 vocabulary learning (Crosson and Moore, 2017). Seventy-five percent of academic words in English are Latinate (Lubliner and Hiebert, 2011), with their main semantic components being bound roots. The present study draws upon Crosson and McKeown’s (2016) terminology, applying the term “Latin root” to roots, most often from Latin, that are the semantic basis of English words but are not free-standing words in English. We apply the term “root-related words” to freestanding words that share a Latin root (e.g., *fluent*, *flush*, *fluid* and *influence* are all root-related words, as all contain the Latin root *flu*).

Latin roots are often the core unit of a word, such as *bene* in *benefit*. If a learner does not know the meaning of the root *bene* (good or well), knowing the derivational affix *dis* may be useless. Thus, knowledge of Latin roots is essential for morphological analysis. Root-related words share phonemes, graphemes, and semantics and thus demonstrate consistent recurring relations among sound, spelling, and meaning. Identification and segmentation of root-related words may contribute to more effective acquisition of word pronunciation, spelling, and reading comprehension.

### Current Study

The current study explored how children learning ESL best learn words through explicit embodied instruction by testing three conditions: writing the words (hand writing roots; HR), manipulating a chunk consisting of letters by dragging it to make up a word (dragging roots; DR), and demonstrating the meanings of words *via* gestures (gesturing roots; GR). The current study aims to address the extent to which these embodied approaches to L2 vocabulary learning contribute to high-quality lexical representations. We examined these three embodied learning conditions in comparison to a control, word-meaning only (WMO) condition. We conducted explicit morphological training to investigate how children learning ESL use morphological information from Latin roots to enhance their vocabulary learning.

The primary research questions of the present study were*:* (1) Whether or not embodied morphological training enhances the quality of lexical representations for novel words in children learning ESL? (2) How/to what extent embodied morphological training instruction differs in enhancing the quality of lexical representations for novel words in children learning ESL due to different aspects of embodied engagement? In relation to these two questions, we predicted that: (1) morphological training using Latin roots will improve word learning by strengthening semantic and orthographic representations of words; (2) hand writing Latin roots will enhance morphological awareness and establish form-meaning associations, facilitating the use of Latin roots to infer the meanings of unfamiliar words; (3) embodied morphological manipulative strategies will support word learning *via* morphological training with Latin roots.

## Materials and Methods

### Participants

One hundred Chinese-speaking children in grades 3 through 6 learning ESL were recruited for participation in the present study. All participants attended public elementary schools in Ningbo City, Zhejiang Province, Southeastern China and had been studying English since grade 3, when they were an average of 9 years old. Participants were randomly assigned to four training conditions in equal proportions: HR, chunking and DR, GR, and word meaning only (WMO; control condition). There were no significant differences in age between groups (*p* > 0.01). English language proficiency was assessed using the Peabody Picture Vocabulary Test (PPVT) and a standardized reading measure before training. There was no statistically significant difference in language proficiency between the groups as measured by these two assessments (*p*s > 0.05). Questionnaires on the children’s Chinese and English learning background (Li et al., 2021) were also completed by their teachers, confirming that students had no learning difficulties in either L1 or L2.

### Training Materials

Eighteen Latin roots and 44 words containing those roots were selected from the Corpus of Contemporary American English (COCA, Davies, 2008). The selected roots comprised four independent Latin roots having no variant and seven roots with one variant. The frequency rank for the selected words ranged from 257 (*receive*) to 56715 (*biped*). There were 18 (=4 + 7*2) sets of trained roots and their 44 corresponding words.

The Latin roots tested in the present study were selected mainly based on frequency, family size, and the sub-categories to which they belonged. All 18 Latin roots and 44 words were taught in each of four learning conditions. The following criteria guided the selection of Latin roots: (1) the most frequent morphographs in English according to Becker et al. (1980) corpus of morphographic units in the 26,000 highest frequency words in English (Davies, 2008); (2) having as many root-related words as possible, such as *exclaim* and *proclaim*. In order to collect more root-related words, low-frequency words could also be selected if they were of shorter length (e.g., *biped* contains only five letters, but ranks 56715 in COCA); (3) belonging to one of the following five sub-categories: body action, body part, mind and emotion, substance, or abstract concept.

Local school teachers were also asked to rate the familiarity of all 44 selected words to guarantee that the target words were all unfamiliar to the students, as highly frequent encounters with any morphological roots could impact whether and how quickly that root would be recognized in other words (Nagy and Hiebert, 2011).

### Training Conditions and Sessions

**FIGURE 1:**
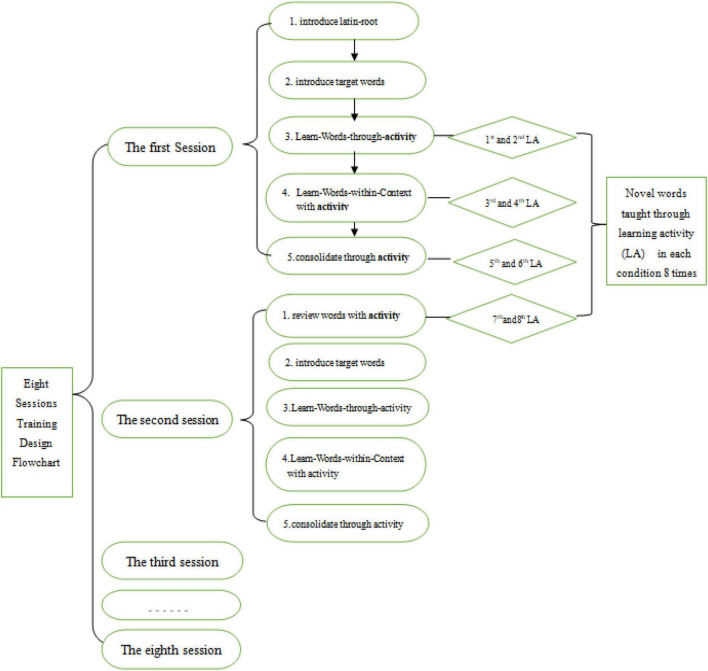
Eight day training flowchart.

All training was conducted in class by four different instructors. The instructors were four researchers who received systematic training in all facets of the project. All four conditions (HR, DR, GR, and WMO) lasted 8 days and covered 18 Latin roots and 60 words. The training materials for all learning conditions followed the design flowchart (see Figure 1) and were identical except for the learning activity. While the learning activities in each training condition differed, each target word was presented a total of eight times during the activity and review in each condition. Figure 1 presents the instruction flowchart and 8 days of learning conditions for each word taught in the four conditions. Each target word was presented six times in the main session when the novel words were introduced through the instructor’s presentation of slides and the learning condition (i.e., hand writing, dragging, gesture, or memorizing the meaning). Participants were exposed to each target word two times in the follow-up session, which began by reviewing the words taught in the previous session.

In the *HR group:* Participants were told to write each root by hand in the blank (e.g., write *grad* in the blank of “____uate”). Each root was written eight times in total.

In the *DR condition*: Participants were instructed to place a sticker printed with the root in the corresponding word blank. For example, on Day 1, “*grad” and “gress”* were printed on four stickers, and participants placed “*grad*” or “*gress*” onto “_____uate,” “up_____,” “re_____” or “ag____sive.” This was repeated eight times for each word.

In the *GR condition*: Participants were presented with a video showing a gesture of the root meaning first and then told to produce the same gesture as the video showed. Videos were made in advance with an experienced native English speaking ESL teacher acting out the 18 roots individually. After playing the video, the instructor acted out each of these gestures again and asked the students to do the same eight times for each word in total. For example, *grad* was gestured as two hands climbing steps, and students copied this gesture eight times.

In the *WMO condition*: Participants visually read the words and memorized the word meaning from the definition eight times.

### Training Procedure

As shown in Figure 1, two Latin roots and their four corresponding new words were introduced in Day 1. For Day 1, there were four steps of training. In *Step 1*, all instructors in each of the four conditions taught the concept of Latin roots briefly by giving an example and explaining their function. In *Step 2*, instructors introduced the new words by prompting participants to guess the meaning of the target Latin roots of the day and confirmed the correct meaning of the root in both Chinese and English. In *Step 3*, instructors conducted the experimental learning conditions (i.e., HR, DR, GR, WMO) in the training session for each word. In *Step 4*, instructors introduced the words used in context and led participants in reading context-based sentences containing the novel words, completing learning activities twice for each of the novel words. In *Step 5*, instructors consolidated the meaning of the words by reinforcing the same learning activities in each representative condition twice. On Day 2, instructors began by reviewing the words taught on the previous day, asking participants to complete the learning activities twice. Therefore, participants in each of the four learning conditions received a total of eight exposures to the target words.

### Measures

Data were collected using both qualitative and quantitative measures. Pre- and post-tests for each of the four conditions were conducted before and after the whole training session, respectively. Participants’ classroom teachers and two trained research assistants administrated a computerized lexical decision task (20-min duration) and a paper-and-pencil word knowledge test (60-min duration) in a formal classroom setting. There were two versions in which the order of the 10 assessments were counterbalanced. Participants were assigned randomly to either of the two versions. All 10 assessments were divided into four categories based on the degree of association between lexical constituents.

#### Category I Tasks: Accessibility to Word Form

##### Lexical Decision (3 Min)

This was an individualized computerized task. The goal of this task was to assess sensitivity to word forms. The stimuli included 48 pseudowords, 24 taught words, and 24 novel words (similar to words used in the Slash-Control Task) controlled for frequency, part of speech, and semantic neighborhood. Pseudowords had been matched on bigram frequency and word length (Martens and de Jong, 2008). The stimuli were presented in E-Prime, and participants completed the task in a quiet computer room. The task instructions were given in Chinese to make sure all participants understood the task requirements. Participants were required to respond as quickly as possible by pressing the “Yes” button if the word was a real word or the “No” button if it was a non-word. The reliability of this task was *a* = 0.87 for all participants.

##### Word Generation Task (15 Min)

This was a group-administered paper-and-pencil test. The goal of this task was to assess participants’ ability to generate words containing the target Latin roots. Fourteen Latin roots were provided. Participants were asked to generate as many words using the given root as they could from their knowledge within 14 min. One minute per root was provided to complete the task.

##### Slash Task for Target Words (1.5 Min)

This was a group-administered paper-and-pencil test aimed at assessing participants’ ability to use a slash to correctly separate letter strings into words. Words taught in training were presented without spaces, four in a row, in random order. Participants were asked to separate the letters in each string into four distinct words. For example, the following string might be presented: “surviveevidencesuperiornegate.” The correct response after slashing separated the string into the words “survive/evidence/superior/negate.”

##### Slash Task for Control Words (1.5 Min)

This task served as a control baseline for the Slash Task for Target Words and was designed to assess participants’ ability to separate letter strings into familiar control words. It was also a group administered paper-and-pencil test. The requirements were the same as the Slash Task for Target Words, except that the words selected were familiar before training.

#### Category II Tasks: Word Form-Sound Association

##### Dictation Task (5 Min)

This task was a group-administered paper-and-pencil test. The classroom teacher recited all 44 words taught during the previous training session. Participants listened to the pronunciation and wrote down the words on a piece of paper. A total of 1.5 points were given for each word (1 point for correct spelling of the root, 0.5 point for correct spelling of the other portion of the word), with 36 points as a full mark for this task. If a portion of the root of the word was provided, it was scored based upon the proportion of accuracy out of 1 point for a correct spelling of that root.

#### Category III Tasks: Word Form-Meaning Association

##### Word Form-Meaning Matching Task (8 Min)

This was a group-administered paper and pencil test designed to evaluate participants’ ability to map a word form onto its meaning. Participants were asked to match the word with its corresponding meaning, which was represented by the word’s definition in English. There were three sets within the word-definition matching task, with eight words and their corresponding definitions comprising one set. A total 24 word-definition pairs were assessed. All the words were taught during the earlier training session.

##### Root Match Task (3 Min)

This was a group-administered paper and pencil test. The goal was to evaluate whether participants had learned the meaning of the Latin roots taught in the training. Participants matched Latin roots with their corresponding meanings. There were three sets within the root-meaning matching task, with six Latin roots and their corresponding meanings comprising one set. A total of 18 roots taught in the training sessions were assessed.

##### Translation Task (5 Min)

This was a group-administered paper and pencil test. The goal was to assess whether participants could recall the meanings of words taught during the training session. They were required to write the Chinese meaning of each English word taught in training. All 44 words were assessed.

#### Category IV Tasks: Morphological Awareness Control

##### Chinese Morphological Awareness Task 1 (12 Min)

This was an individualized task. This morphological compound task (Leong et al., 2008) contained two parts that varied in generating left-headed or right-headed two-character morphological compound words with eight base items each. Participants could choose any six base forms to produce as many “right-headed” two-character words as they could in 6 min. Then, they were asked to choose any six base forms to produce as many “left-headed” two-character words as they could in 6 min. For example, given one of the base forms “马” (ma3, horse), students were required to come up with as many compound words as possible, such as 马上 (literal translation: on the top of the horse = *immediately*), 马路 (literal translation: the path for horse riding = *road*), 马头 (*horse’s head*), 马车 (literal translation *cart driven by the horse*), etc. Two research assistants scored freely affixed items according to the base character. Inter-rater reliability was 0.97. Cronbach’s alpha internal consistency reliability of all the items for this measure was 0.70.

##### Chinese Morphological Awareness Task 2 (5 Min)

This was an individualized morphological chain task. Participants were required to provide as many two-character compound words from the left-headed base character as possible in 5 min. For example, after the first compound word “苹果 (literal translation: apple *fruit = apple*)” was given, the following morphological chain could be produced by the students “果园 (literal translation: fruit yard = *orchid*),” “园艺 (yard *arts*),” “艺人 (artistic *person*),” “苹果 (person’s *heart*),” “心眼 (heart *hole*),” etc. Two research assistants scored the freely affixed items according to the base of the character. Inter-rater reliability was 0.98. Cronbach’s alpha internal consistency reliability of all the items for this measure was 0.74.

### Procedure

Immediately before and after each training session, all participants were assessed using the 10 tasks described above. After the pre-test, participants were randomly assigned to one of four training groups. Independent-samples *t*-tests showed no statistically significant differences in pretest performance on any measures between the four training groups (*p*s > 0.05). Training was conducted for 8 days, with one 45-min session per day.

### Data Analysis

We analyzed our data using linear-mixed effects models (Baayen et al., 2008; Davies et al., 2017), which can simultaneously account for both participant and item level differences. In mixed-effects models, the unit of analysis is the outcome of an individual trial rather than the average across multiple trials. We examined the dependent measure of eight tasks: the accuracy of each of these eight measures, using a generalized mixed-effects model as the log odds (*logit*) of correctly judging a word, log-transformed to reduce positive skew.

We analyzed participants’ true responses in a mixed-effect logit models as a function of two fixed effects^1^ : test times (pre vs. post tests, “Learning” in the formula), and condition (“Cond” in the formula). All variables were coded with mean-centered contrast to obtain estimates of main effects analogous to those from a repeated measures ANOVA.


log(y)ijk=γ+0000γ(Cond-ijCond¯)1000+γ2000(Cond-ijCond¯)+2γL3000earning+kγ13000(Condij−Cond¯)Learningk+γ23000(Condij−Cond¯)2Learning+kγM4000A+ijγ(Cond-ijCond¯)14000MAij+γ(Cond-ijCond¯)24000M2A+iju+ij0v+0j0w+00keijk

where *u*_*ij*0_ is the random intercept for subject *i* (independently sampled from a normal distribution of subject effects with mean 0 and variance ${\text{τ}}_{\text{U}}^{2}$), *v*_0_*_*j*_*_0_ is the random intercept for classroom *j* (independently sampled from a normal distribution of classroom effects with mean 0 and variance ${\text{τ}}_{\text{V}}^{2}$), *w*_00_*_*k*_* is the random intercept for item *k* (independently sampled from a normal distribution of item effects with mean 0 and variance ${\text{τ}}_{\text{W}}^{2}$), and *e*_*ijk*_ is a random trial-level error term (independently sampled from a normal distribution with mean 0 and variance ${\text{σ}}_{e}^{2}$). The model of logit accuracy for subject *i* in classroom *j* responding to item *k* was the same except that the trial-level error term was omitted and the dependent measure was *log* ($\frac{y_{ijk}}{1-y_{ijk}}$), where *y*_*ikj*_ is the probability of subject *i* in classroom *j* responding correctly to item *k*.

**FIGURE 2:**
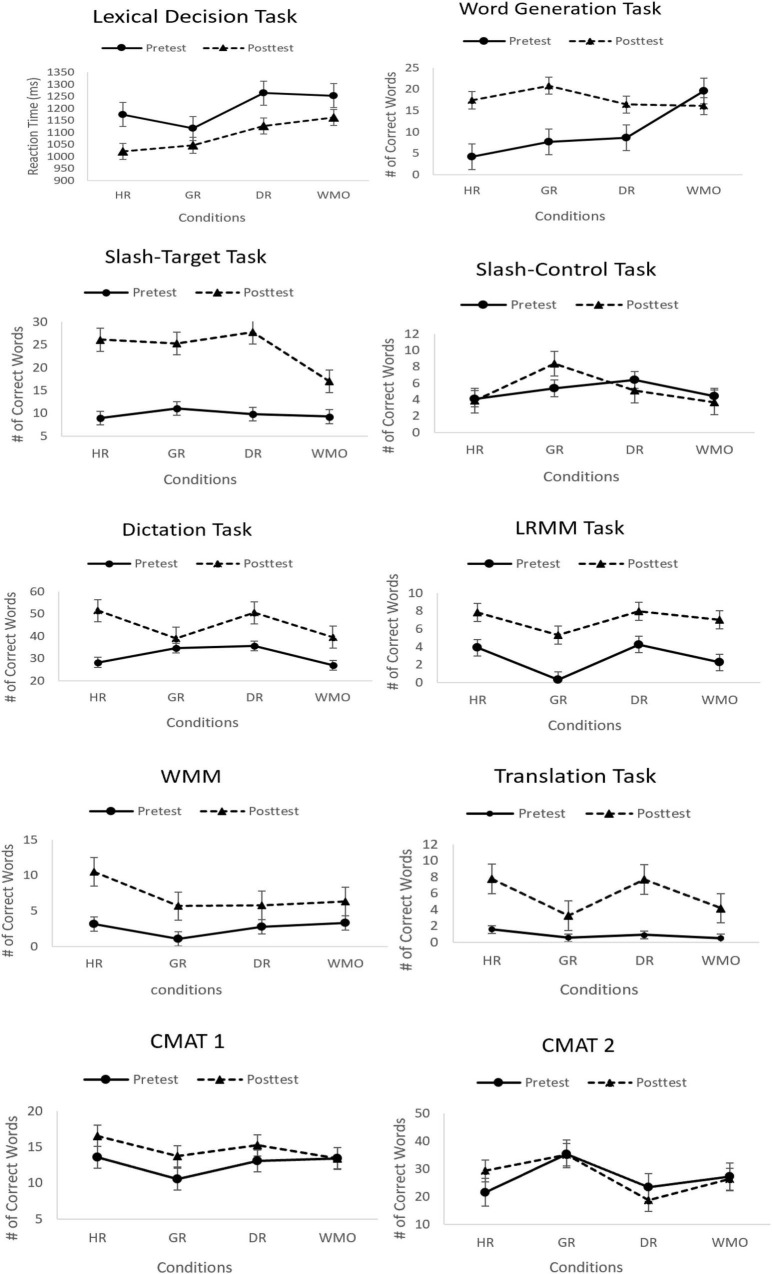
Model-predicted accuracy for eight measures as a function of the partial effects of item-level (pre vs. post) and participant level (four conditions) properties. Error bars depict 95% confidence intervals across subjects. HR, handwrite root; GR, gesture root; DR, dragging root; WMO, word meaning only; LRMM, latin root meaning-matching; WMM, word meaning-matching; CMAT, Chinese morphological awareness task.

The four ordered conditions (HR, handwrite root; GR, gesture root; DR, chunking and dragging root; WMO, word meaning only) were coded using Helmert contrast, which compares each successive condition group to the mean of the other condition group (see Figure 2 with the mean of rates of correct responses for each condition group).

In all models, we included both participant, classroom, and item (word) random intercepts to account for both participant differences and, critical to the motivation of the analysis, item differences. We adopted a model-based approach to outlier detection by fitting an initial model, eliminating observations with residuals more than three standard de*via*tions from the mean, then refitting each model (Baayen et al., 2008). This procedure identifies observations that are outliers after considering all fixed and random effects of interest. All models were fit in R using package *lme4* (Bates et al., 2015). Fixed effects were tested using the Wald *z* test for logit models and the Sattherthwaite approximation to the *t* distribution for Gaussian models (package *lmerTest*; Kuznetsova et al., 2017), all with an a = 0.05 criterion for significance.

## Results

The major results of our analysis are presented in the following order. First, the descriptive statistics of all measures in ten assessments between four conditions show the mean differences between conditions (see Table 1). The omnibus test results of main effects of learning and condition, and the learning by condition interaction of all tasks were then reported in Table 2. Second, based on the preplanned hypotheses about the differences between specific condition. We then reported the only significant results in learning effects (pre vs. post) and condition effects (between each pair of conditions) of the linear mixed modeling analysis in ten assessments between four conditions (see Table 3). Third, we summarized the comparisons between conditions in four category tasks in Tables 4, 5. We present the results in three categories: (1) accessibility of word form, (2) sound-form association, and (3) form-meaning association. These results suggest that the lexical quality of word representations formed by participants in each of the three embodied learning conditions as well as the word-meaning control condition differed significantly.

**TABLE 1:** Descriptive statistics of all measures in 10 assessments between four conditions.

	Conditions							
	HR		GR		DR		WMO	
Tasks	*M*	*SD*	*M*	*SD*	*M*	*SD*	*M*	*SD*
**Pretest**								
Lexical Decision	1174.78	355.11	1116.52	207.68	1263.50	262.15	1253.17	248.41
Word Generation	4.16	3.10	7.62	5.10	8.60	5.74	19.54	8.92
Slash-Target	8.93	7.58	11.04	11.93	9.76	10.57	9.22	11.26
Slash-Control	4.10	2.43	5.36	7.29	6.40	7.19	4.40	5.00
Dictation	28.21	8.66	34.54	10.23	35.67	8.59	26.98	13.55
LRMM	3.88	2.44	0.28	0.72	4.24	2.89	2.24	2.05
WMM	3.16	1.91	1.05	1.69	2.76	2.54	3.28	2.05
Translation	1.54	2.85	0.52	0.74	0.88	1.09	0.48	0.77
CMAT 1	13.56	4.78	10.52	5.65	13.08	6.66	13.44	5.36
CMAT2	21.44	9.26	35.36	1.78	23.32	10.71	27.08	10.28
**Posttest**								
Lexical Decision	1021.66	333.18	1045.96	222.25	1127.87	194.08	1162.58	171.02
Word Generation	17.40	12.72	20.81	6.32	16.40	9.51	16.04	8.29
Slash-Target	26.08	22.11	25.26	22.50	27.70	22.07	16.98	16.17
Slash-Control	3.88	3.71	8.36	6.75	5.12	4.50	3.64	3.95
Dictation	51.46	19.78	39.02	13.13	50.49	12.78	39.51	15.63
LRMM	7.84	4.20	5.29	3.19	7.96	5.95	7.00	3.06
WMM	10.48	5.18	5.67	4.82	5.76	4.58	6.32	3.12
Translation	7.76	5.49	3.24	3.35	7.68	6.44	4.16	3.79
CMAT 1	16.56	7.33	13.71	3.07	15.24	5.42	13.40	3.18
CMAT2	29.24	8.66	35.10	5.20	18.64	6.76	26.24	8.62

*HR, handwrite root; GR, gesture root; DR, chunking and dragging root; WMO, word meaning only; LRMM, latin root meaning-matching; WMM, word meaning-matching; CMAT, Chinese morphological awareness task.*

**TABLE 2:** The Omnibus test of ANOVAs results for category tasks.

Category	Measures	Effects	*F*(df)	MSE	*P*	η^2^
I Accessibility of Word Form	Lexical decision	Learning	1.056_(1,100)_	100.872	0.893	0.001
		Condition	0.093_(3,100)_	14.918	0.579	0.019
		Learning × Condition	0.791_(3,100)_	76.98	0.429	0.011
	Word Generation	Learning	44.94_(1,100)_	4928.99	<0.001	0.323
		Condition	5.501_(3,100)_	201.132	0.002	0.149
		Learning × Condition	12.41_(3,100)_	1360.86	<0.001	0.284
	Slash Target	Learning	50.73_(1,100)_	21375.1	<0.001	0.351
		Condition	0.607_(3,100)_	100.041	0.612	0.019
		Learning × Condition	1.78_(3,100)_	751.917	0.015	0.054
II	Dictation	Learning	104.9_(1,100)_	16544.59	<0.001	0.528
		Condition	2.754_(3,100)_	360.219	0.047	0.081
		Learning × Condition	10.70_(3,100)_	1686.207	<0.001	0.255
III. Word Form Meaning	Latin-Root Meaning Match	Learning	73.59_(1,100)_	1338.208	<0.001	0.439
		Condition	10.17_(3,100)_	60.214	<0.001	0.245
		Learning × Condition	0.093_(3,100)_	16.918	0.429	0.029
	Word-form Meaning Match	Learning	66.56_(1,100)_	1579.725	<0.001	0.415
		Condition	8.617_(3,100)_	51.421	<0.001	0.216
		Learning × Condition	3.558_(3,100)_	84.440	0.017	0.102
	Translation	Learning	86.791_(1,100)_	1973.325	<0.001	0.480
		Condition	6.414_(3,100)_	47.906	0.001	0.170
		Learning × Condition	4.161_(3,100)_	94.596	0.008	0.117

**TABLE 3:** Significant fixed effects estimates from mixed Logit model of correctness with pre-planned comparisons of condition as fixed effects for five tasks.

Task	Effects		Estimate	*SE*	Wald *z*	*p*-value
Lexical decision	Condition Effect	HR vs. WMO	,−0.60	0.06	–10.81	<0.001
		DR vs. WMO	–0.70	0.06	–12.62	<0.001
Word Generation	Condition Effect	HR vs. WMO	0.29	0.11	5.59	<0.001
		DR vs. WMO	–0.78	0.11	–10.81	<0.001
LRMM	Learning Effect	Pre vs. Post	–0.52	0.04	–2.04	0.04
	Condition Effect	GR vs. WMO	–0.34	0.11	–2.64	0.01
		GR vs. WMO	–0.68	0.15	–4.66	<0.001
		GR vs. DR	–0.34	0.15	–2.24	0.02
WMM	Condition Effect	GR vs. HR	0.29	0.06	5.59	<0.001
		HR vs. DR	–0.56	0.06	–10.11	<0.001
Translation	Condition Effect	WMO vs. HR	0.12	0.07	1.63	0.10
		GR vs. HR	–0.09	0.06	–1.47	0.02
		DR vs. GR	–0.17	0.07	–2.46	<0.001

*HR, handwrite root; GR, gesture root; DR, chunking and dragging root; WMO, word meaning only; LRMM, latin root meaning-matching; WMM, word meaning-matching. Only significant effects are presented in the table. All other condition comparisons are not significant, to safe space, so they are not reported in this table.*

**TABLE 4:** Summary table of simple effect test among conditions.

	Pairwise comparison between conditions						
	Condition	HR	DR	GR	HR	HR	GR
		vs.	vs.	vs.	vs.	vs.	vs.
Tasks		WMO	WMO	WMO	GR	DR	DR
**Pre- vs. posttest**							
Lexical Decision	0.046	—	—	—	—	—	—
Word Generation	0.149*	HR > WMO**	DR > WMO*	—	—	—	—
Slash-Target	0.019	—	—	—	—	—	—
Slash-Control	0.049	—	—	—	—	—	—
Dictation	0.080*	—	—	—	—	—	—
LRMM	0.245***	—	—	GR > WMO*	GR > HR***	—	GR > DR***
WMM	0.215*	—	—	—	GR > HR***	HR > DR**	—
Translation	0.17	WMO > HR*	—	—	GR > HR***	—	DR > GR*
CMAT 1	0.025	—	—	—	—	—	—
CMAT 2	0.405***	—	—	—	—	—	—

HR, handwrite root; GR, gesture root; DR, dragging root; WMO, word meaning only; LRMM, latin root meaning-matching; WMM, word meaning-matching; CMAT, Chinese morphological awareness task. *p < 0.05, **p < 0.01, ***p < 0.001.

**TABLE 5:** Summary of comparisons between conditions in four category tasks.

Category	Condition effect
Accessibility to word form	HR > GR > DR > WMO
Word sound-form association	HR > WMO > DR > GR
Word form-meaning association	GR > HR > DR > WMO
Morphological Awareness Control	HR = GR = DR = WMO

There were no significant differences between each pair of conditions in the pretest (*p*s > 1) (see Table 1 for descriptive statistics). Therefore, our preplanned comparisons between pairs of conditions were really to focus on the posttest performance between conditions. The omnibus test of ANOVAs results of main effects of learning and conditions, and the interaction effects of learning by condition of all category tasks were then reported in Table 2.

### Effects of Learning

First, we examined overall learning tests. The positive intercept term indicates that, overall, participants had improved their performance with the averaged odds 1.33 (95% CI: [1.19, 1.50]) in favor of making more accurate responses toward 1 for each item for all eight measures. Participants responded more accurately to the correct responses more frequently, indicating that they had at least gained their performance overall, regardless of learning conditions. Specifically, the odds of responding true for each item gained from 1.82 to 3.51 times for all tasks. As the major concerns of the research target questions are related to the effects of experimental conditions, we then explored the condition effect further.

### Effects of Condition

What about the effects of different condition? As seen from Table 4, learning condition had no main effect on four tasks (e.g., lexical decision, slash-target, slash-control, and dictation), for all other four tasks, we attempted to summarized the comparisons between conditions in the four categories tasks below (see Table 4).

The results of the mixed-effects modeling suggested that there was some evidence that participants in the HR condition scored performance better on the tasks overall. For the category tasks of *accessibility to word form*, participants corresponded to a significant 1.25 times (95% CI: [1.02, 1.29]) better in the odds of successfully making correct responses in the HR condition than the GR condition, and a marginal 1.13 times (95% CI: [1.01, 1.27]) increase in the GR condition than the DR condition, and 1.09 (95% CI: [1.00, 1.25]) marginally increase in the DR condition than the WMO condition.

For the category tasks of *word sound-form association*, participants corresponded to a significant 1.15 times (95% CI: [1.02, 1.29]) better in the odds of successfully making correct responses in the HR condition than the WMO condition, and a marginal 1.11 times (95% CI: [1.01, 1.24]) better in the WMO condition than the DR condition, and 1.07 (95% CI: [1.00, 1.21]) marginally better in the DR condition than the GR condition.

For the category tasks of *word form-meaning association*, participants corresponded to a significant 1.35 times (95% CI: [1.04, 1.29]) better in the odds of successfully making correct responses in the GR condition than the HR condition, and a marginal 1.23 times (95% CI: [1.03, 1.26]) better in the HR condition than the DR condition, and 1.03 (95% CI: [1.00, 1.12]) marginally better in the DR condition than the WMO condition. For the Morphological Awareness control task, there were no significant difference between conditions. We summarized the performance below in Table 5.

The effect comparison between these four experimental conditions corresponding to four categories of tasks are summarized in Table 5. To summarize these results, HR facilitated sound-meaning integration in all category-tasks for accessibility to word form and one task for word sound-form association in comparison with the WMO condition. By contrast, GR facilitated word from-meaning association in two out of three category tasks in comparison with the WMO condition. Chunking and DR facilitated meaning-based integration in one out of three category tasks in comparison with the GR condition, but its facilitative effect in English vocabulary instruction was significantly better in all three category tasks in comparison with WMO condition.

To be concise, it should be noted all three embodied morphological instruction on roots enhanced L2 vocabulary learning. For condition comparison, HR leads to greater effect on sound-form integration, while GR leads to greater effect on form-meaning integration.

## Discussion

We took three embodied word learning conditions (HR, DR, GR) and compared them to a WMO condition. The aim was to investigate how children learning ESL use morphological information from Latin roots to improve their L2 vocabulary learning. Our findings were threefold: (1) HR facilitated sound-meaning integration; (2) GR facilitated meaning-based learning integration; (3) DR enhanced meaning-based integration.

### Hand Writing Facilitates Sound-Form Meaning Integrated Learning

We found that hand writing facilitates sound-meaning integration. This is compatible with Guan et al.’s (2011) finding that the combination of Chinese handwriting and pinyin together can facilitate learning to read Chinese. Why does HR promote sound-meaning integration? One possible answer is that more listening, speaking, and reading and writing activities in the classroom can strengthen the connection between the phonological forms and meanings of words. In addition, hand writing reflects the fundamental properties of the language system. For skilled learners, activation of the phonological forms of words occurs for all writing systems. Hand writing may lead to the creation of motor representations of spelling patterns that support the development of children’s orthographic knowledge. From an applied perspective, the practice of writing words can help children learn to spell (Pritchard et al., 2021). Hand writing is a constructive self-generated learning process or interaction with the construction of sub-lexical letter units of the words being taught. Hand writing engages students in an active decoding process. When learning a decoding system, hand writing is decoded using the language system. In this hand writing process, the visual and auditory systems resonate along with physical or motor embodiment processes to associate phoneme-word correspondences (Guan et al., 2011, 2015; Mizuochi-Endo et al., 2021).

### Gesturing Roots Facilitates Meaning-Focused Word Learning

Gesturing roots facilitates the integration of meaning-based learning. This is in line with Singer et al.’s (2008) claim that gestures can help people sensitively capture multiple forms of information representations and sort out the relationships between them and their role in reasoning processes and meaning construction. The reason for this may be that gestures can stimulate intrinsic motivation, in turn improving academic performance (Shakroum et al., 2018). Gestures are part of linguistic output and contain various meanings that are conveyed visually and holistically. Understanding the meaning of gestures is a reasoning process. When the meanings of gestures are understood, it entails construction of the meaning of accompanying language (Parrill and Sweetser, 2004). This also validates Dick et al.’s (2012) finding that children exhibit sensitivity to the meanings of gestures. The developing brain processes the meanings of gestures through different patterns of connectivity in the fronto-temporoparietal network. However, gestural training does not engage decoding practices. In the current study, the learning process in the GR condition did not rely on a phoneme-word correspondence system. Gestures, such as the use of “foot” to refer to the Latin root “ped,” did not strengthen the visual and auditory association and their connections to the perceptual-motor system. That is to say, the embodied process of gesturing through pointing the “foot” might not have activated phoneme-word correspondences. Since memory decoding is important for word learning, pointing at “ped” did not access the decoding system. This use of deictic gestures, not the system of language, enters memory. In order to index the meaning of “ped,” more work and effort must be expended to create a new “walking” image that maps to the language learning system to facilitate L2 vocabulary acquisition through gesture (Huang et al., 2019).

### Dragging Roots Facilitates Form-Associated Learning

Dragging roots enhances form-meaning-sound integration in L2 vocabulary learning for Chinese ESL children. This is consistent with Ellis’s (1996) claim that language learners are bound to the orthography and phonology of words. Because it is the word chunks that are dragged, learners need to not only understand the root and word meanings but also use movement to match word components. This chunking and dragging process requires visuomotor integration (Sakai et al., 2003; Bera et al., 2021). Development of visuomotor integration is an important factor in facilitating the development of children’s early literacy skills (Dere, 2019). In language acquisition, one of the primary tasks is segmentation of words embedded in a mostly continuous phonological stream (Saffran et al., 1996). Learning a second language, especially with root-based and chunking approaches, requires learners to be adept at inductive reasoning. Learners need to derive general rules from a large number of specific cases (Miao, 2021).

## Limitations

A few limits of the present study should be taken into consideration when interpreting our findings. First, the learning conditions used were limited in duration to only a few days. The number of Latin roots used was only 20 and was extracted from an integrated corpus. Participants were exclusively Chinese elementary school students recruited from a single province. If appropriate, it is also recommended that future studies consider extracting the corresponding high-frequency roots from national vocabulary syllabi for high schools and universities. Further research conducted in more diverse developmental samples across a longer duration is needed to validate the present findings and promote students’ second language vocabulary learning.

## Conclusion

This study aimed to investigate the most effective type of embodied instruction to promote ESL word learning in Chinese children. Four learning methods (HR, GR, DR, WMO) were tested to investigate whether the three training conditions (HR, DR, and GR) embodying word meaning improve the lexical quality of representations of new words compared to the lexical meaning-only control condition (WMO). The differences between these three learning methods were found in those aspects. The study found three main results. One was that handwritten roots promote sound-meaning integration. Second, GR facilitates meaning-based learning integration. Third, DR enhances the form-meaning-sound integration of L2 vocabulary. The study showed that, in the process of teaching vocabulary based on roots, hand writing, gesturing, and dragging should be emphasized so that form-meaning-sound integration can be achieved, contributing to second language vocabulary learning.

## Data Availability Statement

The raw data supporting the conclusions of this article will be made available by the authors, without undue reservation.

## Ethics Statement

The studies involving human participants were reviewed and approved by University of Science and Technology, Beijing, China. Written informed consent to participate in this study was provided by the participants’ legal guardian/next of kin.

## Author Contributions

CG contributed to the designing, theoretical arguments, data collection, analyses, and writing and editing of the project. Both authors contributed to the article and approved the submitted version.

## Conflict of Interest

The authors declare that the research was conducted in the absence of any commercial or financial relationships that could be construed as a potential conflict of interest.

## Publisher’s Note

All claims expressed in this article are solely those of the authors and do not necessarily represent those of their affiliated organizations, or those of the publisher, the editors and the reviewers. Any product that may be evaluated in this article, or claim that may be made by its manufacturer, is not guaranteed or endorsed by the publisher.

## Footnotes

1 With just four classrooms, the analyses do not intend to get a precise estimate of the variance across classrooms in the population, but to include this random intercept to control for differences across classrooms.

## References

[B1] Allen L. Q. (1995). The Effects of Emblematic Gestures on the Development and Access of Mental Representations of French Expressions. *Mod. Lang. J.* 79 521–529. 10.1111/j.1540-4781.1995.tb05454.x

[B2] Austin E. E. Sweller N. (2014). Presentation and production: the role of gesture in spatial communication. *J. Exp. Child Psychol.* 122 92–103. 10.1016/j.jecp.2013.12.008 24549229

[B3] Baayen R. H. Davidson D. J. Bates D. M. (2008). Mixed-effects modeling with crossed random effects for subjects and items. *J. Mem. Lang.* 59 390–412. 10.1016/j.jml.2007.12.005

[B4] Bates D. Mächler M. Bolker B. Walker S. (2015). Fitting Linear Mixed-Effects Models Using lme4. *J. Stat. Softw.* 67 1–48.

[B5] Becker W. C. Dixon R. Anderson-Inman L. (1980). *Morphographic and Root Word Analysis f 26,000 High Frequency Words. Technical Report 1980-1*. Eugene, OR: University of Oregon Follow Through Project.

[B6] Beilock S. L. Lyons I. M. Mattarella-Micke A. Nusbaum H. C. Small S. L. (2008). Sports experience changes the neural processing of action language. *Proc. Natl. Acad. Sci. U.S.A.* 105 13269–13273. 10.1073/pnas.0803424105 18765806 PMC2527992

[B7] Bera K. Shukla A. Bapi R. S. (2021). Motor Chunking in Internally Guided Sequencing. *Brain Sci.* 11:292. 10.3390/brainsci11030292 33652707 PMC7996945

[B8] Blumenthal-Dramé A. Glauche V. Bormann T. Weiller C. Musso M. Kortmann B. (2017). Frequency and chunking in derived words: a parametric fMRI study. *J. Cogn. Neurosci.* 29 1162–1177. 10.1162/jocn_a_01120 28294712

[B9] Brooks N. Goldin-Meadow S. (2016). Moving to Learn: how Guiding the Hands Can Set the Stage for Learning. *Cogn. Sci.* 40 1831–1849. 10.1111/cogs.12292 26400648

[B10] Buccino G. Mezzadri M. (2015). “Embodied language and the process of language learning and teaching,” in *Emotion in Language: Theory–Research–Application*, ed. Luedtke U. (Amsterdam: John Benjamins Publishing Company), 191–208. 10.1075/ceb.10.10buc

[B11] Cohen P. Adams N. (2001). “An algorithm for segmenting categorical time series into meaningful episodes,” in *International symposium on intelligent data analysis*, eds Hoffmann F. Hand D. J. Adams N. Fisher D. Guimaraes G. (Berlin: Springer), 198–207. 10.1007/3-540-44816-0_20

[B12] Crosson A. C. McKeown M. G. (2016). Middle School Learners’ Use of Latin Roots to Infer the Meaning of Unfamiliar Words. *Cogn. Instr.* 34 148–171. 10.1080/07370008.2016.1145121

[B13] Crosson A. C. Moore D. (2017). When to Take Up Roots: the Effects of Morphology Instruction for Middle School and High School English Learners. *Read. Psychol.* 38 262–288. 10.1080/02702711.2016.1263699

[B14] Dargue N. Sweller N. (2018). Not All Gestures are Created Equal: the Effects of Typical and Atypical Iconic Gestures on Narrative Comprehension. *J. Nonverbal Behav.* 42 327–345. 10.1007/s10919-018-0278-3

[B15] Davies M. (2008). *The Corpus of Contemporary American English (COCA): One billion words, 1990-2019.* Available Online at https://www.english-corpora.org/coca/ (accessed June 23, 2022).

[B16] Davies R. A. Arnell R. Birchenough J. M. Grimmond D. Houlson S. (2017). Reading through the life span: individual differences in psycholinguistic effects. *J. Exper. Psychol. Learn. Memory Cogn.* 43, 1298–1338. 10.1037/xlm0000366 28318285

[B17] De Jong N. H. Groenhout R. Schoonen R. Hulstijn J. H. (2015). Second language fluency: speaking style or proficiency? Correcting measures of second language fluency for first language behavior. *Appl. Psycholinguist.* 36 223–243. 10.1017/s0142716413000210

[B18] De Koning B. B. Tabbers H. K. (2013). Gestures in instructional animations: a helping hand to understanding non-human movements? *Appl. Cogn. Psychol.* 27 683–689. 10.1002/acp.2937

[B19] Dere Z. (2019). Analyzing the Early Literacy Skills and Visual Motor Integration Levels of Kindergarten Students. *J. Educ. Learn.* 8 176–181. 10.5539/jel.v8n2p176

[B20] Dewey J. Authentic I. E. L. (1938). *Experiential Learning.* New Jersey: Pentice Hall.

[B21] Dick A. S. Goldin-Meadow S. Solodkin A. Small S. L. (2012). Gesture in the developing brain. *Dev. Sci.* 15 165–180. 10.1111/j.1467-7687.2011.01100.x 22356173 PMC3515080

[B22] Donker A. Reitsma P. (2007). Young children’s ability to use a computer mouse. *Comput. Educ.* 48 602–617. 10.1016/j.compedu.2005.05.001

[B23] Ehri L. C. (2014). Orthographic mapping in the acquisition of sight word reading, spelling memory, and vocabulary learning. *Sci. Stud. Read.* 18 5–21. 10.1080/10888438.2013.819356

[B24] Ellis N. C. (1996). Sequencing in SLA: phonological memory, chunking, and points of order. *Stud. Second Lang. Acquis.* 18 91–126. 10.1017/s0272263100014698

[B25] Foglia L. Wilson R. A. (2013). Embodied cognition. *Wiley Interdiscip. Rev. Cogn. Sci.* 4 319–325. 10.1002/wcs.1226 26304209

[B26] Franco A. Destrebecqz A. (2012). Chunking or not chunking? How do we find words in artificial language learning? *Adv. Cogn. Psychol.* 8:144. 10.5709/acp-0111-3 PMC3376887 22723813

[B27] Freeman J. B. Dale R. Farmer T. A. (2011). Hand in Motion Reveals Mind in Motion. *Front. Psychol.* 2:59. 10.3389/fpsyg.2011.00059 21687437 PMC3110497

[B28] Glenberg A. M. Witt J. K. Metcalfe J. (2013). From the Revolution to Embodiment. *Perspect. Psychol. Sci.* 8 573–585. 10.1177/1745691613498098 26173215

[B29] Gobet F. Lane P. C. Croker S. Cheng P. C. Jones G. Oliver I. (2001). Chunking mechanisms in human learning. *Trends Cogn. Sci.* 5 236–243. 10.1016/s1364-6613(00)01662-4 11390294

[B30] Goldin-Meadow S. (2011). Learning through gesture. *Wiley Interdiscip. Rev. Cogn. Sci.* 2 595–607. 10.1002/wcs.132 24187604 PMC3813017

[B31] Goldin-Meadow S. Alibali M. W. (2013). Gesture’s role in speaking, learning, and creating language. *Annu. Rev. Psychol.* 64 257–283. 10.1146/annurev-psych-113011-143802 22830562 PMC3642279

[B32] Guan C. Q. Liu Y. Chan D. H. L. Ye F. F. Perfetti C. A. (2011). Writing strengthens orthography and alphabetic-coding strengthens phonology in learning to read Chinese. *J. Educ. Psychol.* 103 509–522. 10.1037/a0023730

[B33] Guan C. Q. Perfetti C. A. Meng W. (2015). Writing quality predicts Chinese learning. *Read. Writ.* 28 763–795. 10.1007/s11145-015-9549-0

[B34] Guan C. Q. Smolen E. R. Meng W. Booth J. R. (2021). Effect of handwriting on visual word recognition in chinese bilingual children and adults. *Front. Psychol.* 12:628160. 10.3389/fpsyg.2021.628160 34122220 PMC8194694

[B35] Gunderson L. D’Silva R. A. (2016). “Second language literacy,” in *Handbook of Research in Second Language Teaching and Learning*, ed. Hinkel E. (Oxfordshire, UK: Routledge), 490–505.

[B36] Hoetjes M. van Maastricht L. (2020). Using Gesture to Facilitate L2 Phoneme Acquisition: the Importance of Gesture and Phoneme Complexity. *Front. Psychol.* 11:575032. 10.3389/fpsyg.2020.575032 33329219 PMC7719629

[B37] Holle H. Gunter T. C. (2007). The role of iconic gestures in speech disambiguation: ERP evidence. *J. Cogn. Neurosci.* 19 1175–1192. 10.1162/jocn.2007.19.7.1175 17583993

[B38] Holler J. Wilkin K. (2011). Co-Speech Gesture Mimicry in the Process of Collaborative Referring During Face-to-Face Dialogue. *J. Nonverbal Behav.* 35 133–153. 10.1007/s10919-011-0105-6

[B39] Hostetter A. B. (2011). When do gestures communicate? A meta-analysis. *Psychol. Bull.* 137 297–315. 10.1037/a0022128 21355631

[B40] Hsiao H. S. Chang C. S. Chen C. J. Wu C. H. Lin C. Y. (2015). The influence of Chinese character handwriting diagnosis and remedial instruction system on learners of Chinese as a foreign language. *Comput. Assist. Lang. Learn.* 28 306–324. 10.1080/09588221.2013.818562

[B41] Huang X. Kim N. Christianson K. (2019). Gesture and vocabulary learning in a second language. *Lang. Learn.* 69 177–197. 10.1111/lang.12326

[B42] Inkpen K. M. (2001). Drag-and-drop versus point-and-click mouse interaction styles for children. *ACM Trans. Comput.–Hum. Interact.* 8 1–33. 10.1145/371127.371146

[B43] James K. H. (2010). Sensori-motor experience leads to changes in visual processing in the developing brain. *Dev. Sci.* 13 279–288. 10.1111/j.1467-7687.2009.00883.x 20136924 PMC4176698

[B44] Johnson-Glenberg M. C. Birchfield D. A. Tolentino L. Koziupa T. (2014). Collaborative embodied learning in mixed reality motion-capture environments: two science studies. *J. Educ. Psychol.* 106 86–104. 10.1037/a0034008

[B45] Kelly S. D. McDevitt T. Esch M. (2009). Brief training with co-speech gesture lends a hand to word learning in a foreign language. *Lang. Cogn. Process.* 24 313–334. 10.1080/01690960802365567

[B46] Kelly S. Healey M. Özyürek A. Holler J. (2015). The processing of speech, gesture, and action during language comprehension. *Psychon. Bull. Rev.* 22 517–523. 10.3758/s13423-014-0681-7 25002252

[B47] Kersey A. J. James K. H. (2013). Brain activation patterns resulting from learning letter forms through active self-production and passive observation in young children. *Front. Psychol.* 4:567. 10.3389/fpsyg.2013.00567 24069007 PMC3780305

[B48] Kontra C. Goldin-Meadow S. Beilock S. L. (2012). Embodied learning across the life span. *Top. Cogn. Sci.* 4 731–739. 10.1111/j.1756-8765.2012.01221.x 22961943 PMC3634974

[B49] Kuznetsova A. Brockhoff P. Christensen H. B. (2017). lmerTest package: tests in linear mixed effects models. *J. Stat. Softw.* 82 1–26.

[B50] Leong C. K. Tse S. K. Loh K. Y. Hau K. T. (2008). Text comprehension in Chinese children: relative contribution of verbal working memory, pseudoword reading, rapid automatized naming, and onset-rime phonological segmentation. *J. Educ. Psychol.* 100 135–149. 10.1037/0022-0663.100.1.135

[B51] Li J. X. James K. H. (2016). Handwriting generates variable visual output to facilitate symbol learning. *J. Exp. Psychol. Gen.* 145:298. 10.1037/xge0000134 26726913 PMC4755885

[B52] Li P. Xi X. Baills F. Prieto P. (2021). Training non-native aspirated plosives with hand gestures: learners’ gesture performance matters. *Lang. Cogn. Neurosci.* 36 1313–1328. 10.1080/23273798.2021.1937663

[B53] Lindgren R. Tscholl M. Wang S. Johnson E. (2016). Enhancing learning and engagement through embodied interaction within a mixed reality simulation. *Comput. Educ.* 95 174–187. 10.1016/j.compedu.2016.01.001

[B54] Lorenz D. Tizón-Couto D. (2019). Chunking or predicting–frequency information and reduction in the perception of multi-word sequences. *Cogn. Linguist.* 30 751–784. 10.1515/cog-2017-0138

[B55] Lubliner S. Hiebert E. H. (2011). An Analysis of English–Spanish Cognates as a Source of General Academic Language. *Biling. Res. J.* 34 76–93. 10.1080/15235882.2011.568589

[B56] Luo J. Niki K. Knoblich G. (2006). Perceptual contributions to problem solving: chunk decomposition of Chinese characters. *Brain Res. Bull.* 70 430–443. 10.1016/j.brainresbull.2006.07.005 17027779

[B57] Macedonia M. (2014). Bringing back the body into the mind: gestures enhance word learning in foreign language. *Front. Psychol.* 5:1467. 10.3389/fpsyg.2014.01467 25538671 PMC4260465

[B58] Martens V. E. G. de Jong P. F. (2008). Effects of repeated reading on the length effect in word and pseudoword reading. *J. Res. Read*. 31, 40–54. 10.1111/j.1467-9817.2007.00360.x

[B59] Mauranen A. (2009). Chunking in ELF: expressions for managing interaction. *Intercult. Pragmat.* 6 217–233. 10.1515/iprg.2009.012

[B60] McCutchen D. Stull S. (2015). Morphological awareness and children’s writing: accuracy, error, and invention. *Read. Writ.* 28 271–289. 10.1007/s11145-014-9524-1 25663748 PMC4314952

[B61] McCutchen D. Logan B. Biangardi-Orpe U. (2009). Making Meaning: children’s Sensitivity to Morphological Information During Word Reading. *Read. Res. Q.* 44 360–376. 10.1598/rrq.44.4.4

[B62] Miao C. (2021). The Analysis and Research on the Root-Based and Chunking Approach—Based on the Theory of Multiple Intelligences. *Front. Educ. Res.* 4 100–108. 10.25236/FER.2021.040719

[B63] Mizuochi-Endo T. Itou K. Makuuchi M. Kato B. Ikeda K. Nakamura K. (2021). Graphomotor memory in Exner’s area enhances word learning in the blind. *Commun. Biol.* 4:443. 10.1038/s42003-021-01971-z 33824412 PMC8024258

[B64] Morett L. M. (2014). When Hands Speak Louder Than Words: the Role of Gesture in the Communication, Encoding, and Recall of Words in a Novel Second Language. *Mod. Lang. J.* 98 834–853. 10.1111/modl.12125

[B65] Morett L. M. (2018). In hand and in mind: effects of gesture production and viewing on second language word learning. *Appl. Psycholinguist.* 39 355–381. 10.1017/s0142716417000388

[B66] Morett L. M. Chang L.-Y. (2014). Emphasising sound and meaning: pitch gestures enhance Mandarin lexical tone acquisition. *Lang. Cogn. Neurosci.* 30 347–353. 10.1080/23273798.2014.923105

[B67] Nagy W. E. Hiebert E. H. (2011). “Toward a Theory of Word Selection,” in *Handbook of Reading Research, Volume IV*, eds Kamil M. L. Pearson P. D. Moje E. B. Afflerbach P. P. (Milton Park: Routledge), 414–430. 10.4324/9780203840412.ch17

[B68] Parrill F. Sweetser E. (2004). What we mean by meaning: conceptual integration in gesture analysis and transcription. *Gesture* 4 197–219. 10.1075/gest.4.2.05par 33486653

[B69] Perfetti C. A. Harris L. N. (2013). Universal reading processes are modulated by language and writing system. *Lang. Learn. Dev.* 9 296–316. 10.1080/15475441.2013.813828

[B70] Perruchet P. Poulin-Charronnat B. Tillmann B. Peereman R. (2014). New evidence for chunk-based models in word segmentation. *Acta Psychol.* 149 1–8. 10.1016/j.actpsy.2014.01.015 24632521

[B71] Pouw W. T. J. L. de Nooijer J. A. van Gog T. Zwaan R. A. Paas F. (2014). Toward a more embedded/extended perspective on the cognitive function of gestures. *Front. Psychol.* 5:359. 10.3389/fpsyg.2014.00359 24795687 PMC4006024

[B72] Pritchard V. E. Malone S. A. Hulme C. (2021). Early handwriting ability predicts the growth of children’s spelling, but not reading, skills. *Sci. Stud. Read.* 25 304–318. 10.1080/10888438.2020.1778705

[B73] Pulvermüller F. (2005). Brain mechanisms linking language and action. *Nat. Rev. Neurosci.* 6 576–582. 10.1038/nrn1706 15959465

[B74] Pulvermüller F. Fadiga L. (2010). Active perception: sensorimotor circuits as a cortical basis for language. *Nat. Rev. Neurosci.* 11 351–360. 10.1038/nrn2811 20383203

[B75] Reese H. W. (2011). The learning-by-doing principle. *Behav. Dev. Bull.* 17 1–19. 10.1037/h0100597

[B76] Saffran J. R. Newport E. L. Aslin R. N. (1996). Word segmentation: the role of distributional cues. *J. Mem. Lang.* 35 606–621. 10.1006/jmla.1996.0032

[B77] Sakai K. Kitaguchi K. Hikosaka O. (2003). Chunking during human visuomotor sequence learning. *Exp. Brain Res*. 152 229–242. 12879170 10.1007/s00221-003-1548-8

[B78] Schreuder R. Baayen R. H. (1995). “Modeling morphological processing,” in *Morphological Aspects of Language Processing*, ed. Feldman L. B. (Mahwah, NJ: Lawrence Erlbaum Associates, Inc), 257–294. 10.1002/0470018860.s00254

[B79] Shakroum M. Wong K. W. Fung C. C. (2018). The influence of gesture-based learning system (GBLS) on learning outcomes. *Comput. Educ.* 117 75–101. 10.1016/j.compedu.2017.10.002

[B80] Singer M. Radinsky J. Goldman S. R. (2008). The role of gesture in meaning construction. *Discourse Process.* 45 365–386. 10.1080/01638530802145601

[B81] Singson M. Mahony D. Mann V. (2000). The relation between reading ability and morphological skills: evidence from derivational suffixes. *Read. Writ.* 12 219–252. 10.1023/a:1008196330239

[B82] Skulmowski A. Rey G. D. (2018). Embodied learning: introducing a taxonomy based on bodily engagement and task integration. *Cogn. Res. Princ. Implic.* 3:6. 10.1186/s41235-018-0092-9 29541685 PMC5840215

[B83] Spivey M. J. Grosjean M. Knoblich G. (2005). Continuous attraction toward phonological competitors. *Proc. Natl. Acad. Sci. U.S.A.* 102 10393–10398. 10.1073/pnas.0503903102 15985550 PMC1177386

[B84] Stafura J. Z. Perfetti C. A. (2014). Word-to-text integration: message level and lexical level influences in ERPs. *Neuropsychologia* 64 41–53. 10.1016/j.neuropsychologia.2014.09.012 25234645 PMC4362931

[B85] Stennett R. G. Smythe P. C. Hardy M. (1973). Visual perception of word chunks and beginning reading. *Can. J. Behav. Sci.* 5 280–289. 10.1037/h0082353

[B86] Tellier M. (2008). The effect of gestures on second language memorisation by young children. *Gesture* 8 219–235. 10.1075/gest.8.2.06tel 33486653

[B87] Thalmann M. Souza A. S. Oberauer K. (2019). How does chunking help working memory? *J. Exp. Psychol. Learn. Mem. Cogn.* 45:37. 10.1037/xlm0000578 29698045

[B88] Vukovic N. Shtyrov Y. (2014). Cortical motor systems are involved in second-language comprehension: evidence from rapid mu-rhythm desynchronisation. *NeuroImage* 102 695–703. 10.1016/j.neuroimage.2014.08.039 25175538

[B89] Wakefield E. M. James K. H. (2015). Effects of learning with gesture on children’s understanding of a new language concept. *Dev. Psychol.* 51 1105–1114. 10.1037/a0039471 26214229

[B90] Wiley R. W. Rapp B. (2021). The effects of handwriting experience on literacy learning. *Psychol. Sci.* 32 1086–1103. 10.1177/0956797621993111 34184564 PMC8641140

[B91] Zemlock D. Vinci-Booher S. James K. H. (2018). Visual–motor symbol production facilitates letter recognition in young children. *Read. Writ.* 31 1255–1271. 10.1007/s11145-018-9831-z

[B92] Zhen A. Van Hedger S. Heald S. Goldin-Meadow S. Tian X. (2019). Manual directional gestures facilitate cross-modal perceptual learning. *Cognition* 187 178–187. 10.1016/j.cognition.2019.03.004 30877849

